# Lipothioureas as Lipids for Gene Transfection: A Review

**DOI:** 10.3390/ph4101381

**Published:** 2011-10-24

**Authors:** Marie Breton, Jeanne Leblond, Isabelle Tranchant, Daniel Scherman, Michel Bessodes, Jean Herscovici, Nathalie Mignet

**Affiliations:** 1 UMR 8203, Laboratoire de Vectorologie et Thérapeutiques Anticancéreuses, Université Paris-Sud, 91405 Orsay, CNRS Institut Gustave Roussy, 94805 Villejuif, France; 2 Faculté de Pharmacie, Université de Montréal, CP 6128, Succ Centre-Ville, Montréal, QC, H3C 3J7, Canada; 3 Commissariat à l'Energie Atomique, Service d'Ingénierie des Protéines, 152, CE-Saclay, 91191 Gif sur Yvette, France; 4 UMR 8151 CNRS, U1022 INSERM, Unité de Pharmacologie Chimique et Génétique et d'Imagerie, Université Paris Descartes, Sorbonne Paris Cité, Chimie-ParisTech, 4 avenue de l'observatoire, 75006 Paris, France

**Keywords:** lipothiourea, thiourea lipids, DNA, lipoplexes, transfection, gene therapy

## Abstract

Non-viral gene therapy requires innovative strategies to achieve higher transfection efficacy. A few years ago, our group proposed bioinspired lipids whose interaction with DNA was not based on ionic interactions, but on hydrogen bonds. We thus developed lipids bearing a thiourea head which allowed an interaction with DNA phosphates through hydrogen bonds. After a proof of concept with a lipid bearing three thiourea functions, a molecular and cellular screening was performed by varying all parts of the lipids: the hydrophobic anchor, the spacer, the linker, and the thiourea head. Two lipothiourea-based structures were identified as highly efficient *in vitro* transfecting agents. The lipothioureas were shown to reduce non specific interactions with cell membranes and deliver their DNA content intracellularly more efficiently, as compared to cationic lipoplexes. These lipids could deliver siRNA efficiently and allowed specific cell targeting *in vitro*. *In vivo*, thiourea lipoplexes presented a longer retention time in the blood and less accumulation in the lungs after an intravenous injection in mice. They also induced luciferase gene expression in muscle and tumor after local administration in mice. Therefore, these novel lipoplexes represent an excellent alternative to cationic lipoplexes as transfecting agents. In this review we will focus on the structure activity studies that permitted the identification of the two most efficient thiourea lipids.

## 1. Introduction

Non-viral gene therapy is an attractive strategy to provide gene transfection while avoiding the safety concerns raised by viral vectors. Non-viral vectors are synthetic compound**s** which can be produced on a large scale, can transport a high payload of plasmids of different size, and can also be repeatedly injected thanks to their low immunogenicity. Non-viral vectors have been investigated particularly against the cystic fibrosis disease which has allowed progress on both transporters and plasmids [1,2]. More recently, interest in non-viral vectors for cardiovascular diseases was also reported [3]. Concerning oncology, progress is still awaited in gene therapy to deliver DNA, siRNA and miRNA to achieve specific treatments [4].

Most gene delivery systems use electrostatic interactions to complex DNA and exhibit a cationic character. Unfortunately, this positive charge enhances non-specific interactions and therefore, cationic vectors cannot be strictly envisioned to specifically target tissues. This problem has usually been dealt with by masking the cationic surface charge [5], but rarely by engineering non-charged vehicles [6]. The few reported examples involve highly original systems such as spherulites [7], nucleolipids [8-10] or saccharidic clusters [11].

In our search for an alternative system, we envisioned that a new approach based on hydrogen bonding could provide gene protection and reduce unspecific interactions. Indeed, the tertiary structure of biomacromolecules is based on different types of non-covalent interactions, including hydrogen bonds. Although hydrogen bonds are weak, they are able to maintain a complex tridimensional structure, as evidenced by the double-stranded helix structure of DNA. We hypothesized that the multi-hydrogen bonding provided by a supramolecular assembly would induce a cooperative effect leading to DNA condensation. For this purpose, we chose the thiourea function, known to be the strongest neutral hydrogen bond donor to carboxylates and phosphates.

The thiourea function is similar to a urea function, in which the oxygen atom has been replaced by a sulphur atom. The two amino groups surrounding the C=S linkage strengthen the electronegativity of the sulphur atom, enhancing its capacity to form hydrogen bonds [12]. Thiourea functions even allow the formation of solids maintained solely by intermolecular linkages [13]. As it was first considered for anion recognition purposes, the thiourea function has been more thoroughly studied in organic solvents and in extreme pH conditions than in aqueous media and physiological pH. From the available data, the function appears to be chemically stable and little affected under physiological conditions [14,15]. Moreover, complexation of phosphate anions in water showed the superiority of thioureas as compared to ureas [16]. Thioureas were also shown to facilitate lipid membrane crossing of complexed anions [17,18].

Considering the above mentioned advantageous properties of the thiourea moiety, we therefore chose to incorporate this function at the polar head of a lipid, in order to form lipothiourea-based liposomes [19]. We could show that the thiourea function maintained its property of interacting with phosphates in a lipidic form. Afterwards, amphiphilic cationic cyclodextrins based vectors bearing aminothiourea segments have also been reported, showing the participation of the thiourea segments in DNA complexation through cooperative electrostatic and hydrogen bonding interactions [20-22]. More recently, mannosylated mixed thiourea and cationic DNA cyclodextrin complexes were shown to be internalised specifically by macrophages [23].

For the purpose of this review, we will focus on neutral lipothioureas, for which interactions with negatively charged serum proteins and proteoglycans are expected to be greatly reduced. This review summarises the evolution of these lipids from the first prototype to the current well-characterized and biologically active lipothiourea lipids.

## 2. Results and Discussion

### 2.1. Thiourea Lipid Structure Optimization

#### 2.1.1. Proof of Concept with the Trithiourea Lipid

**Figure 1:**
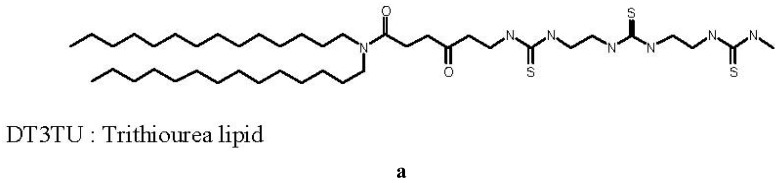

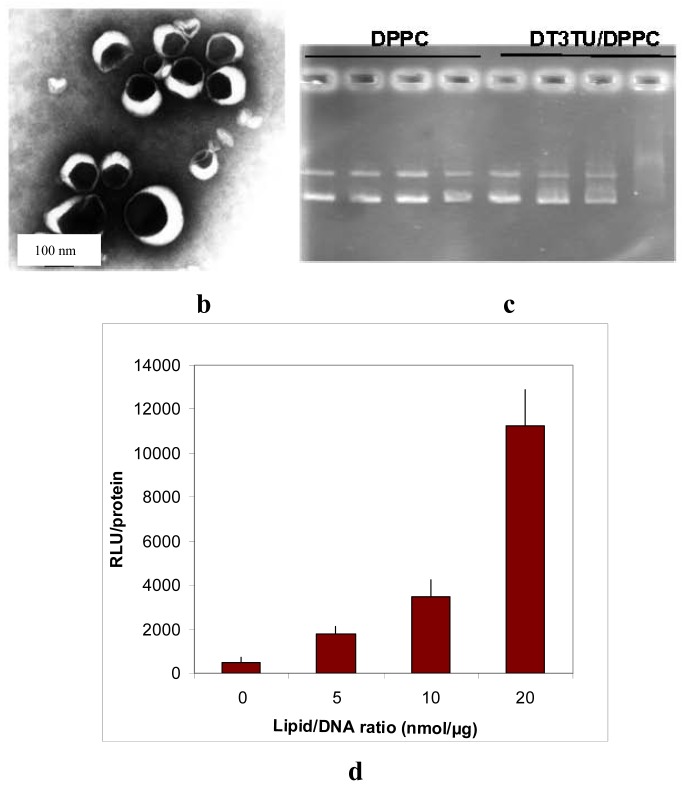
(**a**) The firstly synthetized lipothiourea. DT3TU is linear and bears three thiourea functions; (**b**) Formulated with DPPC, it forms a suspension of spherical colloids of 100 nm size, as observed by TEM; and (**c**) associates DNA at a ratio of 20 nmol of lipid/μg DNA or 17 lipid/DNA equivalent weight. From left to right, the wells were loaded with free DNA, DPPC/DNA at a ratio 5, 10, 20 nmol of lipid/μg DNA, free DNA, DT3TU/DPPC/DNA at a ratio 5, 10, 20 nmol of DT3TU lipid/μg DNA; (**d**) DT3TU transfects Hela cells in a dose dependent manner as shown with a luciferase gene reporter gene The relative light unit measured is normalised by the amount of protein in μg in each well. DT3TU/DPPC/DNA lipoplexes were evaluated at the ratios 5, 10, 20 nmol of lipid/μg DNA.

The first thiourea-based lipid synthesized, called DT3TU, bore three thiourea functions, as shown in Figure 1. Its design was inspired by the cationic lipid RPR120535, previously designed in our laboratory, which exhibited a high transfection efficacy [24]. The polar head of the lipothiourea was linear, with three thiourea functions available for an interaction with DNA phosphates. However, unlike its cationic counterpart, it was not amphiphilic and thus hardly suspended in water.

Despite this major drawback, DT3TU could be formulated with dipalmytoylphosphatidylcholine (DPPC) or PEG-lipid to avoid aggregation, and obtain a formal proof of concept of the maintained thiourea function capacity to interact with DNA in its lipidic form [19]. When associated to DT3TU, DNA was protected from DNAse-mediated degradation [19], and transfection of HeLa cells was achieved (Figure 1). More importantly, circulation time of the lipothiourea nanoparticles in mouse blood was comparable to the circulation time of conventional liposomes. Altogether, these results were sufficiently encouraging to lead us to further conceive novel lipothiourea molecules for gene delivery [25].

#### 2.1.2. Screening Dithiourea Lipids Structures in Terms of Amphiphilicity

**Figure 2:**
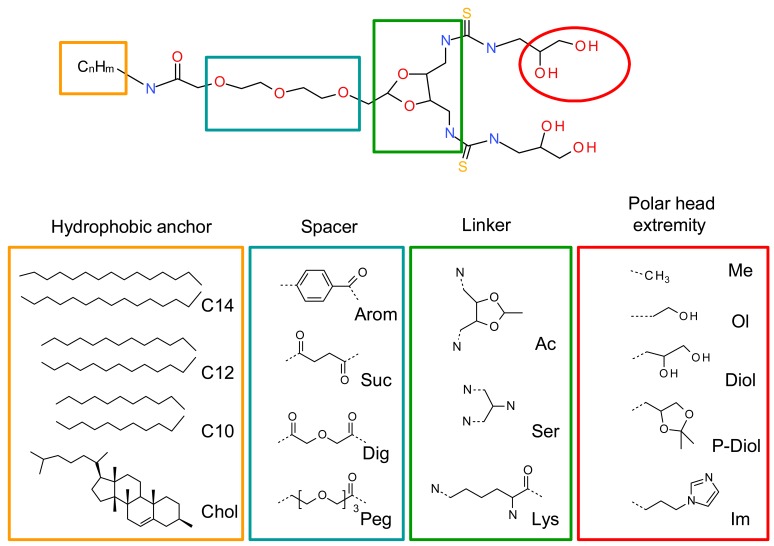
Screening of the different parts of the lipothiourea to optimise the amphiphilic balance as well as the transfection efficiency. The hydrophobic anchor was varied from aliphatic chains (C10, C12, C14) to a cholesterol residue. Aromatic, succinic, diglycolic and ethylene glycol spacers were tested. Acetal, serinol and lysine-based linker were compared. The extremity of the polar head was substituted either by a methyl, an alcohol, a protected or unprotected diol and an imidazole.

A systematic structure-activity relationship was performed to establish the crucial structural points of the new lipids. The four lipid components, namely the hydrophobic anchor, spacer, linker and polar head extremities were thoroughly evaluated (Figure 2). The geometry of the polar headgroup was modified to obtain an optimal branching structure based on the literature data, and on our results with compound RPR209120, also called DMAPAP [5,26-28]. Different lipid chain lengths were proposed as this factor was previously shown to have an impact on transfection efficiency [29]. Various spacers were also synthesized, such as aromatic, succinic, diglycolic or ethylene glycol to evaluate their impact on the dispersion of the lipid in an aqueous medium. The linker part was also modulated to provide different orientation and distance between the two thiourea functions and evaluate its effect on DNA condensation [30]. Finally, various polar head extremity was synthesized to ensure amphiphilicity to the lipid construct and eventually favour the formation of hydrogen bonds with the surrounding media and/or DNA. The structures obtained are summarised in Figure 2.

We first evaluated the solubility of the compounds in an aqueous medium, in ethanol or in chloroform, to choose the appropriate method to form a suspension with these lipids in an aqueous medium, either hydration of a lipidic film or ethanolic injection. LogP was calculated for all the lipids, as summarised in Figure 3. LogP represents the octanol/water partition coefficient which in turn reflects the amphiphilic property of a lipid. Comparing the solubility of the lipids in water and their logP values, we found that the structures exhibiting a logP between 3.5 and 4.8 presented an optimised hydrophilic/lipophilic balance and could be obtained as nanometric particles in an aqueous medium. The lipids which presented a logP above 6.3 could not form nanoparticles in water and had to be suspended in presence of dipalmytoylphosphatidylcholine (DPPC), as for the lysine series. When the association of this colipid did not help suspending the thiourea lipids, the candidate molecule was discarded from further studies, as for the aromatic and succinic series.

#### 2.1.3. Chemical Synthesis of the Selected Compounds

**Scheme 1:**
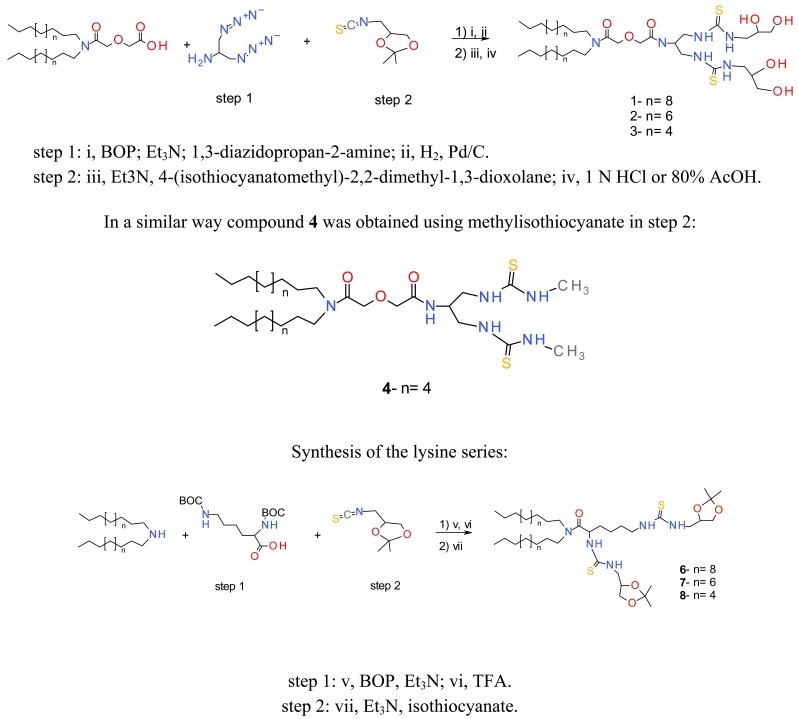
General synthetic scheme of the biologically tested compounds. The first series is based on a serinol linker (**1–4**), the second series is based on a lysine linker (**6–8**). The hydrophobic anchor was varied from C_10_ to C_14_ in the two series, except for compound **4** which was obtained by a route similar to the first series, except for step 2, where methylisocyanate was used instead of the isopropylidene isocyanate derivative.

The selected compounds have been obtained on a larger scale thanks to an optimised chemical process. The general synthesis is described in Scheme 1. Briefly, the dialkylamines were reacted with diglycolic anhydride in dichloromethane to give the corresponding carboxylic lipids. 1,3-diazidopropan-2-amine was obtained in three steps from serinol [31]. Condensation of the two fragments was then performed using classical peptide bond formation procedures. Benzotriazole-1-yl-oxy-tris-(dimethylamino)phosphonium hexafluorophosphate (BOP) was chosen as the activator to facilitate the purification of the product. The azide groups were then hydrogenated over palladium on carbon catalyst to give the corresponding diamine derivatives. The isothiocyanate used in step 2 was synthesized as described earlier, starting from commercially available (2,2-dimethyl-1,3-dioxolan-4-yl)methanamine [31]. The amino function of this compound was reacted with carbon disulfide in the presence of dicyclohexylcarbodiimide, the obtained reagent could be distilled with a Kugelrohr apparatus. This product was reacted with the previously obtained diamines to give the protected lipid thioureas (**5** n = 4; not represented). The isopropylidene protecting groups were then hydrolysed with aqueous HCl to give the final products **1**, **2** and **3**.

To obtain compounds **6–8**, the dialkylamines were condensed with Boc-N-protected lysine using BOP as the activating agent. The protecting groups were subsequently cleaved by the action of trifluoroacetic acid, and the resulting diamino derivatives were reacted with the isothiocyanate to give the protected thioureas **6**, **7** and **8**. It should be pointed out that deprotection of the diol groups was not possible in this series, due to the Edman degradation of the α-*N*-thiourea function [32]. Nevertheless, the corresponding unprotected lipid obtained via a different and tedious route appeared to be less efficient in transfection experiments.

#### 2.1.4. Thiourea Lipid/DNA Interaction

The number of thiourea functions has been reduced from three to two between the first thiourea lipid synthesized and the series of lipids described above (Scheme 1). We showed that a lipid bearing two thiourea functions efficiently interact with DNA, whereas a lipid bearing only one thiourea function was insufficient to condense with DNA [33].

The ability of most of the lipids to interact and associate DNA was evaluated by different methods. Gel retardation assays gave the thiourea/phosphate ratio required to fully retain DNA as indicated in Table 1. However, in a classical test using a base intercalating fluorophore, the level of fluorescence was not reduced by the addition of lipothiourea, indicating that DNA bases were still accessible in these liposomes. To determine the effective thiourea/phosphate ratio involved in a lipothiourea/DNA interaction, we turned to a more sensitive technique optimised by Kral and coworkers [34]. The mobility of a single DNA labelled molecule was followed by fluorescence correlation spectroscopy and analyzed by an autocorrelation function. In this technique, addition of increasing amounts of lipothiourea slows down DNA mobility as soon as an interaction occurs. Using FCS, we evidenced an interaction at a ratio of one thiourea per phosphate and a full condensation at a ratio of 5 nmol lipid/μg DNA for the A_C10_S_Dig_L_Ser_P_diol_ compound (number **3** in Table 1, also called DDSTU in the literature) [35]. Increasing the lipid length from C_10_ to C_14_ (compounds **3**, **2** and **1**), reduced the efficiency of the interaction with DNA for the serinol series. The opposite was found for the lysine series formulated with DPPC which interacted more efficiently with DNA by increasing the lipid length. We will see next that this has a dramatic impact on the transfection efficiency.

This study also evidenced the influence of the chemical function at the polar head extremity. Comparing compounds **3**, **4**, **5** in Table 1, which solely differ by their polar head extremity, we observed that increasing the hydrophilicity (from **5** to **3**), increased the lipothiourea/DNA interaction efficacy. To further study the interaction occurring between DNA and lipothiourea assemblies, we performed a thorough NMR study by synthesizing a ^13^C-enriched compound **3**. This study indicated that the last deprotection step corresponding to the isopropylidene hydrolysis in acidic medium was crucial for this series. Deprotection led to compounds of similar molecular mass, but with either an iminothiol or a thiourea function depending on the strength of the acid used (strong hydrochloric acid or weak acetic acid). The presence in liposomes of the iminothiol which can only form and remain stable in drastic conditions could only be explained by the favoured hydrogen bonds of the lipidic auto-assembly [36].

### 2.2. Physico-Chemical Characterisation of Thiourea Lipid/DNA Particles

Using cryo-transmission electronic microscopy, we showed that lipothioureas self-assembled as unilamellar liposomes following ethanolic injection of low concentration in water Figure 3a. The size distribution was repeatedly measured in the 30–80 nm range by dynamic light scattering. Moreover, using static light, neutron and small angle X-ray scattering, we evidenced that the complexation with DNA induced a structural reorganisation of the liposomes, that led to the formation of multilamellar domains with DNA inserted between the lipid bilayers Figure 3b [37]. Stability experiments demonstrated that the lipoplexes released less than 15% of their DNA content over 48 h in serum at 37 °C. Moreover, lipothiourea protects DNA from enzymatic degradation. As can be seen on agarose gel Figure 3c, DNA was totally degraded after incubation in fresh mouse serum during 24 h, while DNA was protected when complexed with lipoplexes. In addition, more intact DNA was recovered from lipothiourea lipoplexes as compared to cationic lipoplexes Figure 3c, either suggesting a stronger lipid/DNA interaction or a reduced DNAse interaction with the thiourea lipoplexes.

### 2.3. In Vitro Evaluation of Thiourea Lipoplexes

#### 2.3.1. DNA Transfection with Thiourea Lipoplexes

**Figure 4:**
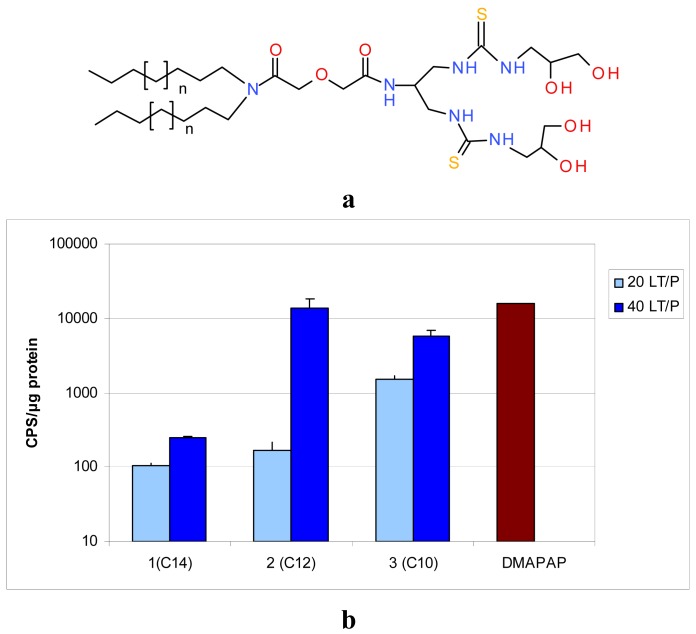

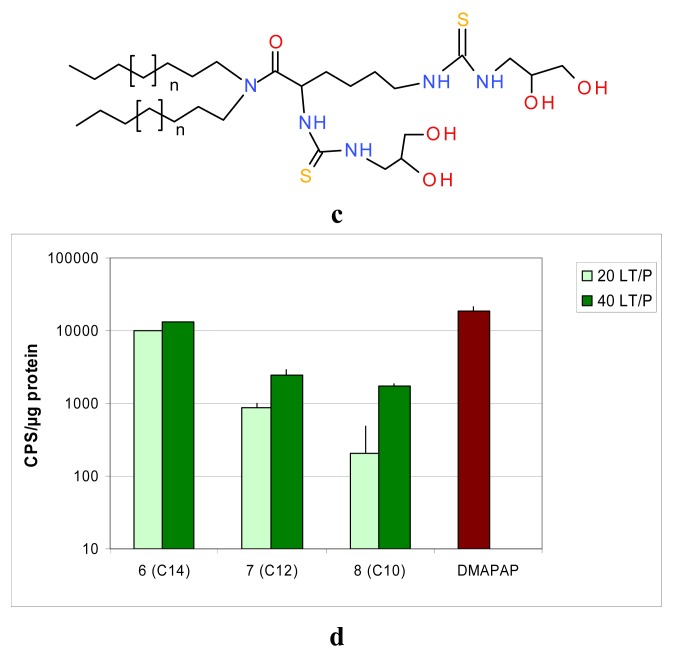
Schematic representation of the two lipothiourea series retained for further studies and example of transfection results obtained on B16 cells after 48 h incubation. (**a**) Derivatives bearing a diglycolic spacer and a serinol linker; (**b**) Transfection of B16 cells at 2 lipid/DNA ratio with lipids **1**, **2**, **3**; (**c**) Derivatives bearing a lysine linker; (**d**) Transfection of B16 cells at two lipid/DNA ratios (20 and 40 nmol lipid/μg DNA) with lipids **6**, **7**, **8**. The cationic lipid DMAPAP was used at a N/P ratio = 4. Transfection efficiency is given in CPS/μg protein representing the signal (counts per second) of the luciferase emission normalised by the amount of protein quantified with the BCA kit. Each bar represents the mean +/− SD of three independent wells. The exact protocol is described in [31] for compounds **1**, **2**, **3**, in reference [38] for compounds **6**, **7**, **8**. No significant difference was found between **2**, **3** at the ratio 40 and DMAPAP using the Mann-Whitney U-test.

Based on these physico-chemical data, the compounds in Table 1 were selected for further tests in terms of cytotoxicity and transfection efficacy *in vitro*. Apart from compound **5**, all lipids tested transfected cells with various efficiencies (Figure 4) [31,38]. Levels of transfection have been compared to our lead compound DMAPAP, which is as efficient as the commercial Lipofectamine™ in the conditions used. There was no significant difference between DMAPAP and lipoplexes made with compounds **3** and **6** under their optimal conditions. As these compounds are non-cationic lipids and do not interact as efficiently with the cell membrane as cationic lipoplexes, as reported in the next paragraph, the fact that lipothiourea gave similar results as cationic lipoplexes was found to be quite a positive result.

Two lipid/DNA ratios (20 and 40) were compared, and we noted a clear dose-dependent increase in transfection for both families of lipids Figures 4b,d. Interestingly, the lipid length had a different impact on the two series. In the serinol series, the efficacy increased when the lipid length decreased from 14 to 10 carbons Figure 4b, whereas, in the lysine series, the converse was noted Figure 4d. This difference between the two series could be due to the fact that the lysine series was co-formulated with the lipid DPPC, which might impact the liposome structure and the level of transfection.

Another point of interest is the relationship between lipid/DNA interaction and transfection efficiency. The two lead candidates, compound **3**, (A_C10_S_Dig_L_Ser_P_diol_), and compound **6** (A_C14_L_Lys_P_diolP_) which were found to be the best transfecting agents from their series, are also the lipids which showed the strongest interaction with DNA [35]. In addition, these lipids were also the ones which interacted at a lower lipid/DNA ratio (see Table 1, red boxes). A lower lipid/DNA ratio required presents the advantage of reducing the amount of lipid needed for transfection, and consequently limiting the cytotoxicity of the lipoplexes.

#### 2.3.2. Application to siRNA Delivery

**Figure 5:**
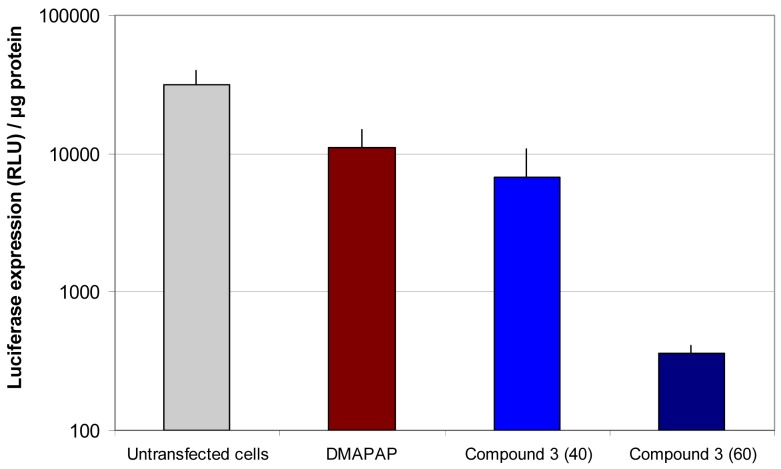
Efficacy of cationic and thiourea lipids to induce reduction of luciferase expression by delivering a siRNA gene silencing luciferase in B16-luciferase positive cell line. Lipid/phosphate ratios used were 40 and 60 nmol lipid/μg siRNA, with 0.15 μg siRNA. Lipoplexes were added in the cell culture medium in presence of 10% foetal calf serum and left 6 h. The medium was changed and the cells left for an additional 24 h before being lysed and processed for luciferase evaluation (for a standard protocol see reference [43]).

In view of the efficacy of lipothiourea **3** to transfect cells with DNA, we thought that this lipid could be applied as a siRNA carrier. We firstly chose to evaluate the feasibility of siRNA delivery by lipothiourea by simply mixing thiourea lipid and siRNA (Figure 5). The ratio between thiourea and phosphates had to be increased as compared to what had been done with DNA, in order to obtain an optimal level of luciferase inhibition. Because siRNA-based lipoplexes are usually less stable than DNA-based lipoplexes, different strategies have been proposed, such as adding complementary anionic charges to cationic liposomes and siRNA [39,40], render siRNA cationic [41] or increasing its size with sticky overhangs [42]. As the feasibility of siRNA delivery by lipothiourea has been shown here, one of these strategies could be applied to increase the efficacy.

#### 2.3.3. *In Vitro* Internalisation of Thiourea Lipoplexes

A quantitative study of labelled DNA internalisation mediated by derivative **3** or its lipoamine counterpart showed that the thiourea lipoplexes were six times less internalised than the cationic lipoplexes made with DMAPAP [44]. This is consistent with a reduced interaction of the thiourea lipoplexes with the negatively charged proteoglycans present at the surface of the cells as compared to the cationic lipoamine lipoplexes. The zeta potential ranges between +30 and +50 mV for lipoamine lipoplexes in NaCl (depending on the N/P ratio), whereas it is between −20 to +20 mV for thiourea lipoplexes [37].

**Figure 6:**
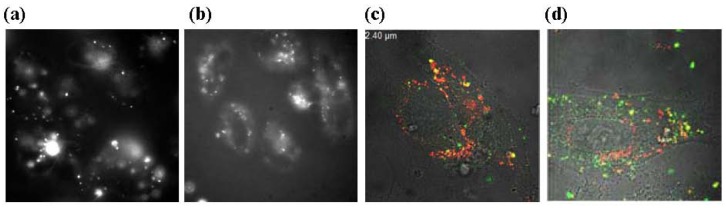
(**a**) Aggregates of rhodamine labeled DNA in cationic lipoplexes at the membrane and internalized by CHO cells in suspension 24 h after incubation; (**b**) Thiourea rhodamine labeled DNA lipoplexes (DDSTU, lipid **3**) internalized as punctuates by CHO cells in suspension 24 h after incubation; (**c**) Cationic lipoplexes labeled with rhodamine (red) and fluorescent DNA (green) internalized by HeLa cells after a 30 minute incubation at 37 °C; (**d**) Thiourea lipoplexes labeled with rhodamine (red) and fluorescent DNA (green) internalized by HeLa cells after a 30 minute incubation at 37 °C.

Following the release of fluorescently labelled DNA into the cells, we also found that thiourea lipids released more efficiently their DNA content [44]. The weaker hydrogen bond based-interaction between lipids and DNA could explain this result of high interest as DNA is mostly trapped and only partly released from the aggregates formed by cationic lipids into the cell. A visual example of this is given in Figure 6 with labelled DNA. Large aggregates are observed for cationic lipoplexes (**a**), whereas in the case of thiourea lipoplexes, a clear membrane localisation is observed (**b**). When both lipids and DNA are labelled, one can clearly observe that DNA (green spots) is more efficiently released from lipothiourea (**d**) than from cationic lipoplexes (**c**). Collectively, these results could explain why complexes, which are poorly internalised by the cells, are transfecting cells efficiently. Thiourea lipoplexes have also been shown to be internalised mostly by a caveolae process while DMAPAP lipoplexes would be mostly internalised via clatrin endocytosis [44]. This difference in the major internalisation pathway could explain the different efficacy of DNA release between thiourea and cationic lipoplexes.

#### 2.3.4. *In Vitro* Targeting with Thiourea Lipoplexes

The initial interest in avoiding cationic charges was to allow cell targeting by reducing unspecific cell interactions. Besides, grafting a targeting ligand at the surface of thiourea liposomes could increase the amount of lipoplexes internalised by specific cells.

**Figure 7:**
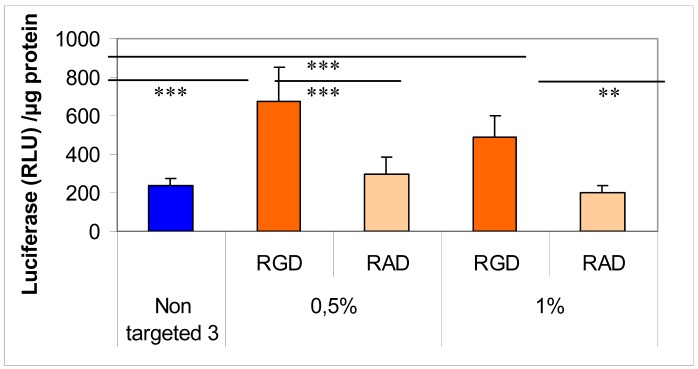
Specific DNA (pCMV-luc plasmid) transfection of a RGD-PEG_2000_-lipid as compared to a RAD-PEG_2000_-lipid incorpored in thiourea liposomes made of compound **3** and to the non targeting pegylated liposomes made with compound **3**. Percentages represent the amount of PEG-lipid incorporated in the liposomes (mol/mol PEG-lipid/total lipid). Complexes were incubated 2 h with EAhy 926 endothelial cells. Relative light Unit (RLU)/μg protein refers to the amount of light measured normalised by the protein amount in each well. Values represent the mean +/− SD of triplicates. Statistics were performed with the Mann- Whitney U-test (*** *p* < 0.001, ** *p* < 0.01).

To this end, we added an α_v_β_3_ integrin ligand by means of a lipid-PEG to target endothelial cells. Addition of a RGD-PEG_2000_-lipid or RAD-PEG_2000_-lipid at the surface of lipothiourea complexes allowed obtaining a higher specific transfection efficiency comparing RGD and RAD ligands as shown in Figure 7 [30]. Unfortunately, targeting integrin induces cell detachment from the plastic support and therefore complicates the transfection experiment. Only a short incubation could be used for these experiments, which explains the low level of transfection and limited differences between the different groups. Moreover, increasing the amount of RGD-PEG_2000_-lipid from 0.5 to 1% decreased the gene expression, probably due to a PEG shielding effect, which could reduce the internalisation of the lipoplexes into the cells [45]. Despite these technical difficulties, conditions could be found to show that the level of transfection was increased only when the RGD was present at the surface of the thiourea lipoplexes.

### 2.4. In Vivo Evaluation of Thiourea Lipoplexes

#### 2.4.1. *In Vivo* Transfection Mediated by Thiourea Lipoplexes

Given the good transfection results obtained with compound **3** *in vitro*, we next investigated the potential application of thiourea lipoplexes *in vivo* in mice. *In vivo*, intramuscular and intratumoral administrations of thiourea lipoplexes have been evaluated using luciferase expression. Thiourea lipids did not inhibit DNA transfection mediated by electrotransfer in the muscle, as found with charged delivery systems [46]. Moreover, the gene transfection and expression by lipothioureas proved to be as efficient as their cationic counterparts in lung carcinoma tumors [31].

#### 2.4.2. *In Vivo* Biodistribution of Thiourea Lipoplexes

The biodistribution of thiourea lipoplexes after intravenous administration was compared to conventional liposomes made with phospholipids. The composition of thiourea liposomes was similar to that of conventional liposomes [47], as they were formed by a mixture of 70% of lipid **3** and 30% of a thiourea-cholesterol derivative. The lysine derivative **6** was tested in presence of DPPC as formulated for in vitro studies.

**Figure 8:**
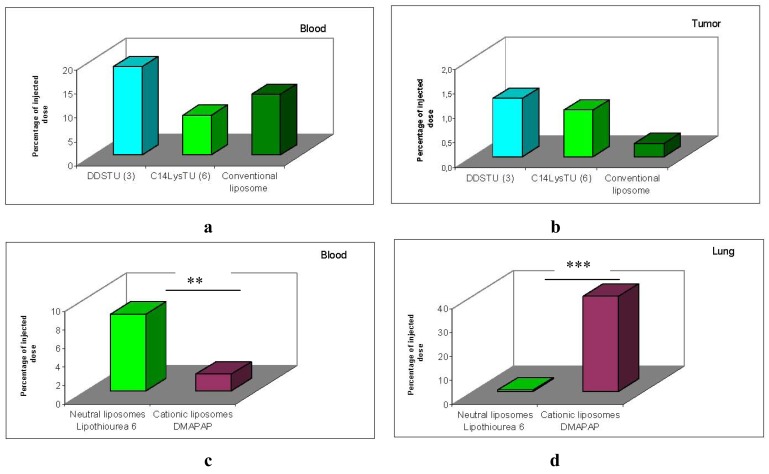
Biodistribution in C57BL\6 mice of rhodamine labelled lipothiourea (LT) **3** and **6** as compared to conventional liposomes in blood (**a**); and 3LL subcutaneous tumors (**b**) 2 h post i.v. injection. Biodistribution in C57BL\6 mice of rhodamine labelled lipothiourea **6** as compared to cationic liposomes in blood (**c**); and in lung (**b**) 2 h post i.v. injection. Labelled lipids were extracted from the blood or the organs and the rhodamine level measured in the extracts as previously reported [38,48] Statistics were performed with the Mann-Whitney U-test (*** *p* < 0.001, ** *p* < 0.01).

Results presented in Figure 8 show that the amount of both thiourea formulations recovered in the blood 2 h post-injection was similar to the amount of neutral conventional liposomes recovered Figure 8a. Moreover, the amount of lipothiourea recovered in tumor was not significantly different from the amount recovered with conventional liposomes Figure 8b. These results confer to the thiourea liposomes a superiority over conventional liposomes which cannot load DNA in absence of divalent cations.

Comparing lipothiourea with cationic lipoplexes, we found that the amount of thiourea lipoplexes was significantly higher in the blood 2 h post-injection Figure 8c. Noteworthy, the level in the lung was significantly decreased with lipothiourea **6** showing the reduction of unspecific interactions. These results confirmed our hypothesis that neutral lipids could be more interesting than charged lipids for in vivo gene delivery.

## 3. Conclusions

In summary, we have designed original neutral transfecting vectors containing thiourea functions. The introduction of two thiourea functions in the lipid allowed the compaction and protection of DNA. A rigorous structural activity relationship has been performed to determine the impact of each part of the lipid. We found that the lipid length and the polar head were the parts which impacted the most both formulation and transfection.

We showed that thiourea lipids were able to transfect cells either with DNA or siRNA *in vitro*, even though lipothiourea were poorly internalised by the cells. An efficient release of the nucleic acids in the cells as well as a major caveolae internalisation pathway could explain the efficacy of this transfection.

Finally, the amount of thiourea lipoplexes recovered in the blood *in vivo* was increased as compared to that of charged systems. The level of transfection was still poor, as obtained with cationic lipoplexes, but targeting could improve transfection *in vitro* and should be evaluated *in vivo*. Circulation time of lipothiourea was similar to the one of conventional liposomes, which are currently clinical use for doxorubicin delivery. This is rather interesting as lipothiourea present the advantageous properties over conventional liposomes to condense, protect and transfer DNA into the cells. Further *in vivo* evaluations on relevant models should be performed with adapted formulations including PEG or cholesterol to further increase the lipidic auto-assembly stability.

## Figures and Tables

**Figure 3:**
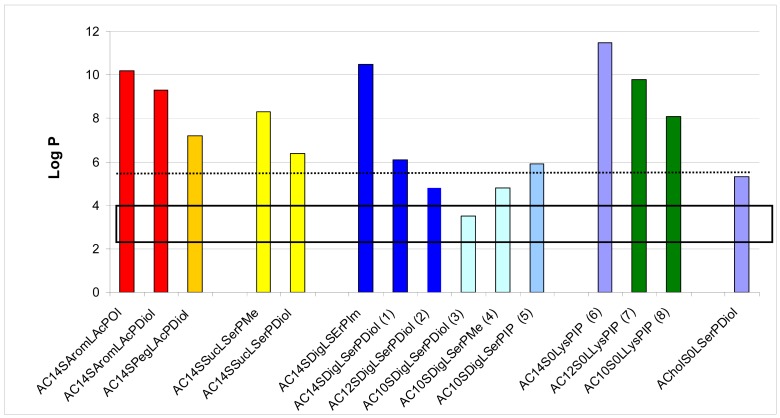
LogP of the different lipothiourea synthesized as calculated with MarvinSketch ^®^ software (ChemAxon, Hungary). LogP between 3.5 and 4.8 (box) were found to be optimised values to formulate thiourea lipids by themselves without addition of energy, just by dilution in water. At a logP below 6.3 (dotted line) the thiourea lipids could still be formulated without addition of a co-lipid. Above 6.3, thiourea lipids required a co-lipid to be suspended in an aqueous medium. The compounds have been named ASLP where A refers to anchor, S spacer, L to linker and P to polar head. As an example, a lipid bearing a 10-carbon chain length, an acetal spacer, a serinol linker and a diol polar head will be named A_C10_S_Ac_L_Ser_P_diol_. A similar lipid which does not bear a Spacer would be called A_C10_S_0_L_Ser_P_diol_.

**Figure 3:**
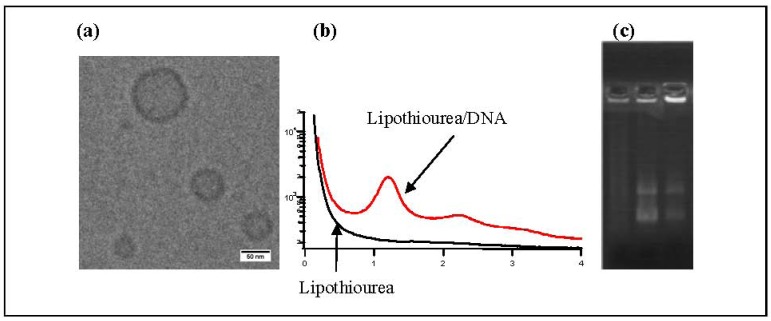
(**a**) Transmission electronic cryo-microscopy of liposomes obtained from compound **3**. Liposome size was 30–80 nm; (**b**) Small Angle X-ray Scattering of lipid **3** without DNA (black curve) in which no order reflexion was observed, or in presence of DNA (red curve) in which a clear second order was observed corresponding to a lamellar phase; (**c**) Incubation of lipoplexes in fresh mouse serum 24 h at 37 °C showed a stabilisation of DNA in presence of lipothiourea. Left lane is DNA (degraded in presence of serum), middle lane is lipothiourea **6**/DNA, right lane is cationic lipid DMAPAP/DNA. DNA was released from the complexes with SDS treatment prior to agarose gel loading.

**Table 1:** Lipothiourea (LPT)/phosphate (nmol/μg) ratio required for a total DNA complexation. DNA interaction was evaluated by two techniques: DNA delayed migration on agarose gel and fluorescence correlation spectroscopy (FCS) [35]. Colours refer to the histograms in Figure 4. ND, not determined. The column + or − co-lipid indicates the formulations which contain or do not contain DPPC. The ratio used between DPPC and the lysine thiourea lipids were 1/1.

**N°**	**Anchor**	**Spacer**	**Linker**	**Polar Head**	**Log P**	**Co-lipid DPPC**	**LPT/P Gel**	**LPT/P FCS**
**1**	C14	Diglycolic	Serinol	diol	6.2	**-**	15	20
**2**	C12	Diglycolic	Serinol	diol	4.6	**-**	15	15
**3**	C10	Diglycolic	Serinol	diol	3.3	**-**	10	5
**4**	C10	Diglycolic	Serinol	Me	4.6	**-**	40	25
**5**	C10	Diglycolic	Serinol	P-diol	5.9	**-**	40	25
**6**	C14	None	Lysine	P-diol	11.5	**+**	5	ND
**7**	C12	None	Lysine	P-diol	9.8	**+**	20	ND
**8**	C10	None	Lysine	P-diol	8.2	**+**	40	ND
